# Using gamification to enhance clinical trial start-up activities

**DOI:** 10.1017/cts.2022.405

**Published:** 2022-05-19

**Authors:** Karen Lane, Ryan Majkowski, Joshua Gruber, Daniel Amirault, Shannon Hillery, Cortney Wieber, Dixie D Thompson, Jacqueline Huvane, Jordan Bridges, E. Paul Ryu, Lindsay M. Eyzaguirre, Marianne Gildea, Richard E. Thompson, Daniel E. Ford, Daniel Hanley

**Affiliations:** 1 Johns Hopkins University School of Medicine, Baltimore, MD, USA; 2 Tufts Medical Center, Boston, MA, USA; 3 University of Utah, Salt Lake City, UT, USA; 4 Duke Clinical Research Institute, Durham, NC, USA

**Keywords:** Gamification, clinical trials, metrics, Trial Innovation Network, trial start-up

## Abstract

**Background:**

The Trial Innovation Network (TIN) is a collaborative initiative within the National Center for Advancing Translational Science (NCATS) Clinical and Translational Science Awards (CTSA) Program. To improve and innovate the conduct of clinical trials, it is exploring the uses of gamification to better engage the trial workforce and improve the efficiencies of trial activities. The gamification structures described in this article are part of a TIN website gamification toolkit, available online to the clinical trial scientific community.

**Methods:**

The game designers used existing electronic trial platforms to gamify the tasks required to meet trial start-up timelines to create friendly competitions. Key indicators and familiar metrics were mapped to scoreboards. Webinars were organized to share and applaud trial and game performance.

**Results:**

Game scores were significantly associated with an increase in achieving start-up milestones in activation, institutional review board (IRB) submission, and IRB approval times, indicating the probability of completing site activation faster by using games. Overall game enjoyment and feelings that the game did not apply too much pressure appeared to be an important moderator of performance in one trial but had little effect on performance in a second.

**Conclusion:**

This retrospective examination of available data from gaming experiences may be a first-of-kind use in clinical trials. There are signals that gaming may accelerate performance and increase enjoyment during the start-up phase of a trial. Isolating the effect of gamification on trial outcomes will depend on a larger sampling from future trials, using well-defined, hypothesis-driven statistical analysis plans.

## Background

Slow site start-up has been a major problem in National Institute of Health (NIH) clinical trials [1,2]. Overcoming the barriers to site activation has become an improvement priority for investigators during the early trial planning stages. Recent benchmarking exercises and metrics are providing early information on specific factors that delay activation at clinical sites. However, it is still early to use site-level start-up data to determine the most reasonable activation timetable and study start-up design.

Compared to performing trial interventions and testing how new approaches work on diseases, trial activation tasks are repetitive and can be uninteresting. Start-up teams must depend on institutional and departmental personnel for approvals and cope with institutional delays and barriers over which they have little control. Traditionally, site teams receive a bundle of trial materials to process and are left on their own to figure out what to do first or in what order or with whom. This results in waste – time and effort – missed deadlines, a sense of isolation, and dissatisfaction. Given most trial teams have more than one trial in motion at any given time, the trial that is in the start-up phase often is placed on the back-burner in favor of more interesting or shifting priorities elsewhere: participant care comes before paperwork.

Start-up involves the usually thankless responsibilities of gathering regulatory approvals, documenting personnel paperwork, and managing others to meet deadlines that are even farther from their minds. Even though the trial science ahead will be innovative and complex, team commitment and engagement can be slow to take form, given the less enjoyable activities of rounding up and filing documents. And through it all, site teams are not engaged with other sites and just beginning to engage with coordinating center staff; they are unaware of how they compare to other sites – whether they are ahead or behind in readiness or if other sites are already enrolling.

There are many opportunities to reverse start-up inefficiencies and delays. Following lean management principles, ordering tasks using a specific flow pathway starting with the tasks that take the longest completion times can help sites do the work faster by working smarter to eliminate downtime and wasted time. And, continuous and regular site engagement can revive teams who might be losing momentum and missing deadlines. Gamifying start-up activities can help with both smart flow and momentum: games can help site teams identify the right pathway and games can engage and motivate site teams by connecting site personnel to other site teams and the coordinating center staff through competition and fun.

Gamification is the application of typical elements of game playing (e.g., point scoring, competition with others, and rules of play) to other areas of activity like the workplace, or in this case, clinical trials, to produce desired effects [3]. It is generally achieved in two ways: the modification of existing activities into a more game-like setting and the addition of structural elements (such as digital badges and leader boards) to encourage a player/user to obtain a goal [4].

Gamification has accumulated popularity as a subject for academic inquiry [5], and successful business models have utilized gamification within the workplace [4]. Although the concept of gamification is becoming more widespread, gamification programs and the gamification of clinical trial performance remain limited. As of this writing, there are no published papers on gamifying clinical trial responsibilities. There are a few known gamification programs to improve trial performance. University of Utah researchers are building a game in collaboration with its Entertainment Arts and Engineering Program to enhance accrual and retention rates. By transforming a site screening log into an e-card game format that recognizes recruitment achievements, this gaming-based approach uses embedded game accolades to incentivize research team members to report accrual metrics. Another game used a World Cup theme: the trial had more than 80 centers around the globe and simulating an international sport was a timely match. The goal of this game was to improve overall trial performance – giving points to boost screening and enrollment ratios and praises for timely follow-up visits and data query responses. The points and weighting system were mapped to the trial’s risk management plan, focusing on fewer study participants lost to follow-up, improved data integrity, and protocol and outcome compliance.

Gaming platforms can be useful for medium-sized, multicenter Phase III clinical trials. Knowing that trial start-up cycles progress slower than expected in most cases and that most sites will be unproductive without engagement and motivation [6], one game was designed to track familiar activation metrics and more frequently engage study coordinators, investigators, and other study staff responsible for completing the trial start-up cycles in two different trials. Although the literature does not yet reflect the potential importance of this technique, there are some signals that engagement with gaming may accelerate start-up cycle time and increase enjoyment and satisfaction, even when the work is repetitive, tangibly unrewarding, and done in isolation.

## Methods

The Johns Hopkins coordinating center developed a gamification platform to implement start-up activities for two trials, titled: The TRaditional versus Early Aggressive Therapy for Multiple Sclerosis (TREAT-MS) and VItamin C, Thiamine and Steroids in Sepsis (VICTAS). Available data on site team satisfaction and speed of site activation were evaluated.

### Building Games into Existing Platforms

The coordinating center (CC) conducted monthly webinars (Go-To-Training®) and used an electronic data collection system (Electronic Data Capture [EDC]; VISION® Prelude LLC, Austin, TX) for trial data; a global electronic management system (GEMS^©^; VISION® Prelude LLC, Austin, TX) for start-up cycle tracking; and an electronic trial management filing system (eTMF; VISION® Prelude LLC, Austin, TX) for document storage. The CC employed centralized site navigators to guide sites through start-up activities that were standardized and staged into sequenced, compartmentalized, one-task-at-a-time objectives to reach firm deadlines. The program was designed to shorten the highest-risk delays: sIRB approval, contract execution, authority delegation, and training. With these platforms, the coordinating center put gamification to work by turning the collective efforts required of site teams during a clinical trial start-up into a friendly competition, with the intent to introduce enjoyment and motivation into tasks that required hard-to-meet start-up timelines and deadlines (each individual site team in a race against the trial clock).

Key start-up indicators (metrics) were collected by the coordinating center and mapped to scoreboards. Core metrics included completion of IRB approvals and contracts, regulatory document completion, and completion of training requirements. Game scoreboards were built into an electronic trial management platform functioning as a start-up tracker (e.g., REDCap) and exported to a dedicated trial website on a monthly basis. Both electronic platforms and the website were available only to the coordinating center and site teams. Scoreboards were accessible to site teams so they could check their site scores in real time and the scores of all sites on the website. Webinar broadcasts to participating sites were organized by the coordinating center to provide a forum where trial and game information was shared and applauded. These broadcasts were central to keeping sites engaged in the games, by showcasing monthly site rankings and providing recognition for accomplishments as a regular feature. Additional coordinating center effort was required to create graphics, present standings, and host awards at monthly webinars or through other means of communication.

**Fig. 1.**
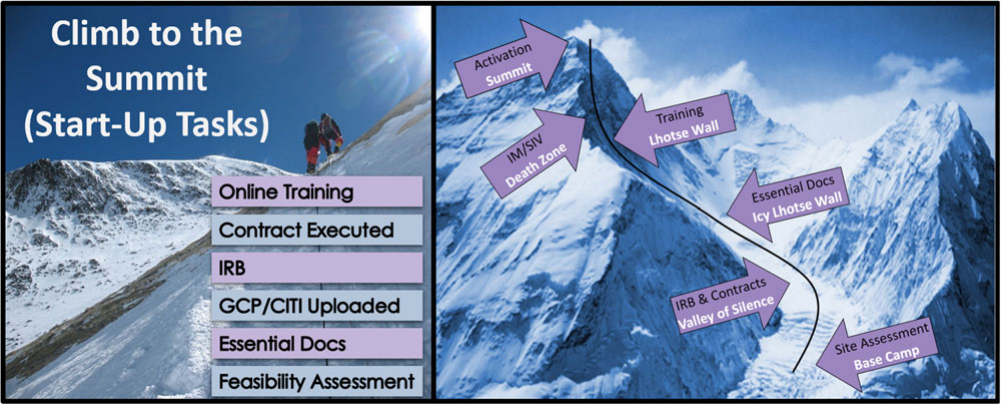
Clinical trial start-up tasks (left panel) were gamified into a Mt. Everest Climb (right panel) to enhance performance during a site activation phase. Monthly task completion was matched to reaching Mt. Everest base camps and the summit.

Elements of game playing (scoring, competition, and rules) were applied by simulating a Mount Everest climb (Fig. 1, left panel). To gamify the full process and not simply score based on which team crossed the finish line first, individual start-up tasks were assigned point values. Once a task was completed, points were awarded and posted on a leaderboard in the eTMF and current standings in reaching higher Mt. Everest basecamps (i.e., matching meaningful, pre-set monthly start-up milestones) and how close sites were to the summit (activation) were shared during monthly webinars. Points were allocated to correspond to specific milestones and mapped to the tracking system. To mitigate any timeline delays institutional offices might have on site performance, the Mt. Everest scoring system was weighted heavily on PI and coordinator tasks – steps external to their direct control, such as the IRB approval (the trials utilized a central single IRB model) and sponsor finalization of the contract, were left out of the scoring. The single component dependent on institutional office speed/performance was the “Partial Execution” of the subaward (Table 1). Direct team responsibilities were valued higher than institutional personnel tasks to complete and were worth more points to win or lose. Likewise, bonuses or penalties for the early/late completion of any given step were capped at 14 days, to prevent any single metric from having an outsized effect on overall score. Site teams received bonus points for time-sensitive tasks and points could be subtracted for work submitted late. Activation was assigned a small point value as a final bonus to reward a generally well-performing site.

**Table 1.** Metrics and point values that made up the core ruleset for the Mt. Everest game

Metric	Goal Duration (Days)	Base Points for Completion	Bonus Points per Day Early/Late (capped at 14 days)
Protocol Available to Local Context Questionnaire (LCQ) First Draft	21	5	0.4
Protocol Available to Site Specific Consent Information (SSCI) First Draft	21	5	0.4
Partially Executed Contract Received by Sponsor	42	10	0.7
Regulatory Document Templates Sent to Delegation Log Circulation	35	5	0.4
Delegation Log Circulation to Finalization	14	5	0.4
Regulatory Document Templates Sent to Site Level Regulatory Documentation Completion (Lab Certs, FDA-1572s, IP and Protocol Signature Pages, etc.)	56	15	0.9
Regulatory Document Templates Sent to Personnel Regulatory Documentation Completion (Human Subjects Certifications, CVs, etc.)	56	10	0.7
Training Available to Training Completion	35	10	0.7
Total Site Activation Duration	90	1	0.2
^*^Weekly Meeting Attendance		1 per meeting	N/A
^‡^Monthly Webinar Attendance		1 per webinar	N/A

^*^,^‡^The Mt. Everest game was conducted in the context of an Accelerated Start-up Program which features weekly check-in meetings with the coordinating center and monthly educational webinars covering important start-up topics.

### Study Aims and Statistical Methods

#### Aims

The primary endpoint focused on identifying if overall game performance (e.g., Mt. Everest score) was related to improved trial start-up metrics, such as time to site activation, IRB submission and approval, training completion, and contract execution. A secondary analysis looked at whether perceptions of the game were correlated to trial start-up performance.

#### Satisfaction survey

Once all sites were activated to enrollment-ready status, site teams were surveyed about their satisfaction with the Mt. Everest game and responses were collated. The survey questions were developed by the game designers, and survey data were collected within a confidential Qualtrics survey (Table 2). Survey respondents could be any member of the site start-up team with instructions that only one survey per site should be submitted. No identifiable private information was collected; respondents to the surveys were asked to provide only the name of the institution represented.

**Table 2.** Voluntary, confidential site survey is distributed following the Mt. Everest start-up competition. Highlighted rows are featured in the analysis

**What is your role at your site?**	□ **Investigator** □ **Coordinator**	***Please respond to the following statements about the Mt. Everest Competition***	
**I enjoyed participating in Mt. Everest.**	**□ Agree** **□ Disagree**	**It helped focus my attention on the start-up activities**	**□ Agree** **□ Disagree**
**What was the greatest source of satisfaction for you?**	**□ Incentives** **□ Recognition** **□ Competition** **□ Working as a team** **□ Other**	**It motivated me to overcome obstacles during start-up**	**□ Agree** **□ Disagree**
**What would you improve about the game?**	**Open Text**	**It increased my pace during the start-up process**	**□ Agree** **□ Disagree**
**What did you dislike about the game?**	**Open Text**	**I liked seeing the game rankings shown at webinars**	**□ Agree** **□ Disagree**
**How did you follow your site’s standings?**	**□ Online** **□ Webinars**	**I felt too much pressure when the webinar displayed the rankings**	**□ Agree** **□ Disagree**
**How often did you follow your site’s standings?**	**□ Daily** **□ Weekly** **□ Monthly** **□ I did not actively follow**	**I felt motivated to improve my standings**	**□ Agree** **□ Disagree**
**If participating again, what would be your preferred method of receiving the Mt. Everest standings?**	□ **Email rankings** **□ Weekly phone calls** **□ Newsletters** **□ Online** **□ Webinars**	**Overall, how would you rate your satisfaction with the Mt. Everest game?**	**0 – 10 Scale, 10 being the highest, 0 being the lowest**

Highlighted rows are featured in the analysis.

Analyses required linking metrics to unique site responses. Responses in the survey were compared to institutional metrics, to look for cross-correlations. Responses to two survey questions, “I enjoyed participating in Mount Everest” (Yes or No) and “I felt too much pressure when the webinar displayed the rankings” (Yes or No), were compared to game scores and component metrics for the respective games.

#### Statistical methods

All analyses were conducted post hoc using data already collected from each trial. Start-up metrics were maintained via a custom clinical trial management system called Global Electronic Management System (GEMS) built on the Prelude Dynamics VISION® EDC platform. Due to different start-up processes and timelines for both TREAT-MS and VICTAS, the range of Everest scores differed greatly between the two trials. Therefore, this study compared the relative performance of sites by normalizing performance within each trial using Everest score z-values. To understand the relationship between the z-value adjusted Everest scores and time to achieving the start-up milestones across both TREAT-MS and VICTAS trials, univariate Cox proportional hazard models were utilized. Hazard ratios with 95% confidence intervals were then calculated from the regression models. Performance differences were assessed by site survey responses for each game. The Wilcoxon rank sum test was used to compare mean performance (Everest score) based on survey questions: “I enjoyed participating in [game name]” and “I felt too much pressure when the webinar displayed the rankings.” For this analysis, responses were included only if a site was identified in the survey response. There were three instances of a 2:1 difference of opinion in the TREAT-MS dataset, two for Q1, and one for Q2. All multi-response instances in the VICTAS dataset were unanimous. For sites with multiple responses, an average site response was calculated and rounded to a “yes” or “no” overall site response (e.g., 2 “yes” and 1 “no” was treated as a “yes” site response). There were no sites in which there was a split response (e.g., 1 yes, 1 no). Statistical significance was determined at the 0.05 alpha level. All data analyses and table and figure generation were conducted in R Statistical Software version 4.1.3 and R Studio version 2022.02.1 build 461.

## Results

**Fig. 2.**
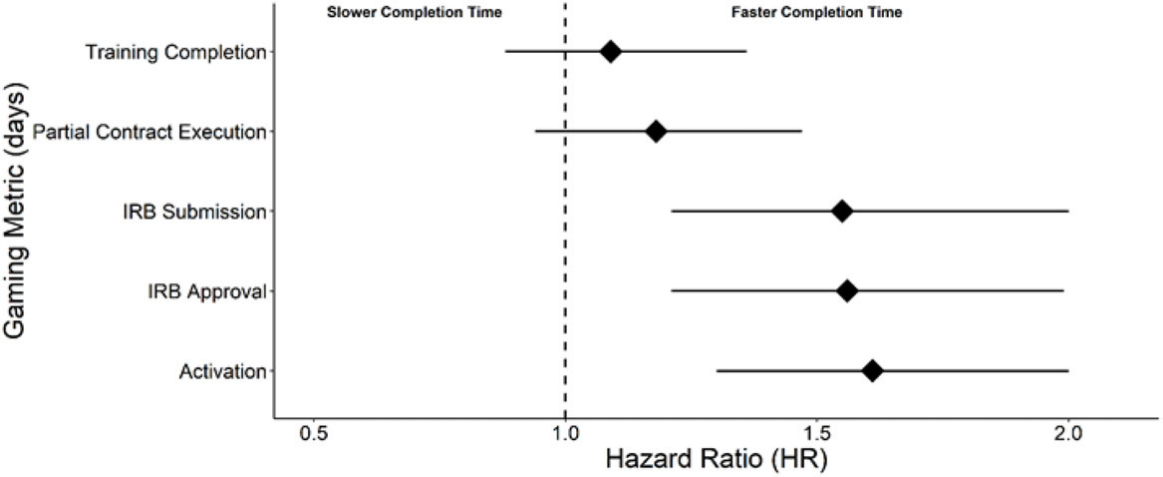
Hazard ratios (HR) for the association between z-value-adjusted Mt. Everest Scores and time to various gaming metrics across both TREAT-MS and VICTAS trials. A higher HR indicates a larger percent increase in the probability of achieving a shorter IRB submission time, IRB approval time, and time to activation.

A higher z-value adjusted Mt. Everest score was significantly associated with an increase in probability of achieving start-up milestones more quickly in three of the five metrics analyzed (activation time, IRB submission time, and IRB approval time) (Fig. 2). Although activation time was the least weighted component of the total Mt. Everest score, it was the most associated metric. This indicates that the probability of completing site activation faster is increased by 61% for every 1 standard deviation increase (or 32 points) in Mt. Everest score.

### Satisfaction Survey Results: Enjoyment, Pressure and Performance

Forty percent (18 of 45) of the TREAT-MS sites and 68% (26 of 38) of the VICTAS sites responded to the satisfaction survey with an identifiable site name. Game participation enjoyment was mixed. In the TREAT-MS trial, 9 of 17 (53%) sites enjoyed participating in Mt. Everest and, in the VICTAS trial, 13 of 25 (52%) sites enjoyed participating in Mt. Everest. In TREAT-MS, 10 of 14 (71%) sites reported not feeling too much pressure when rankings were displayed publicly, and 18 of 23 (78%) sites in VICTAS felt the same.

**Fig. 3.**
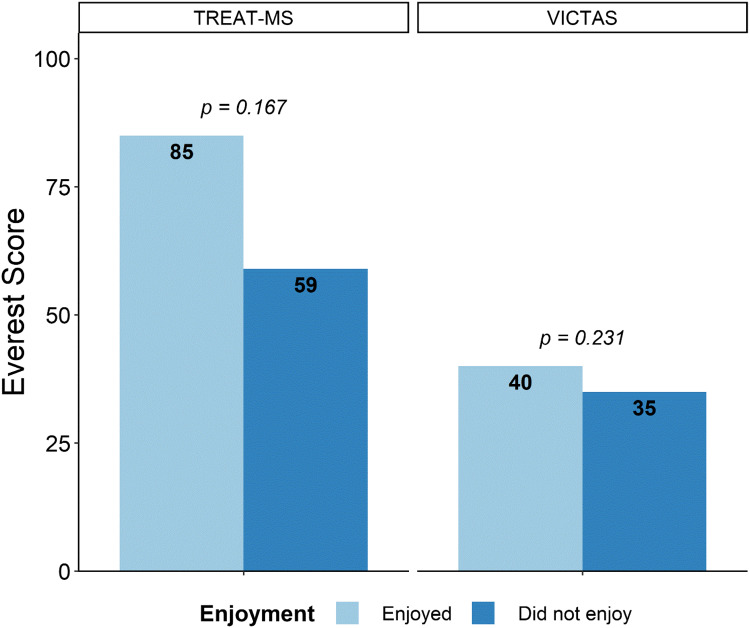
Mean differences in Mt. Everest Score between those who enjoyed or did not enjoy the Mt. Everest game. Enjoyment appeared to be an important moderator of performance in TREAT-MS, but not in VICTAS. Neither result was significant.

**Fig. 4.**
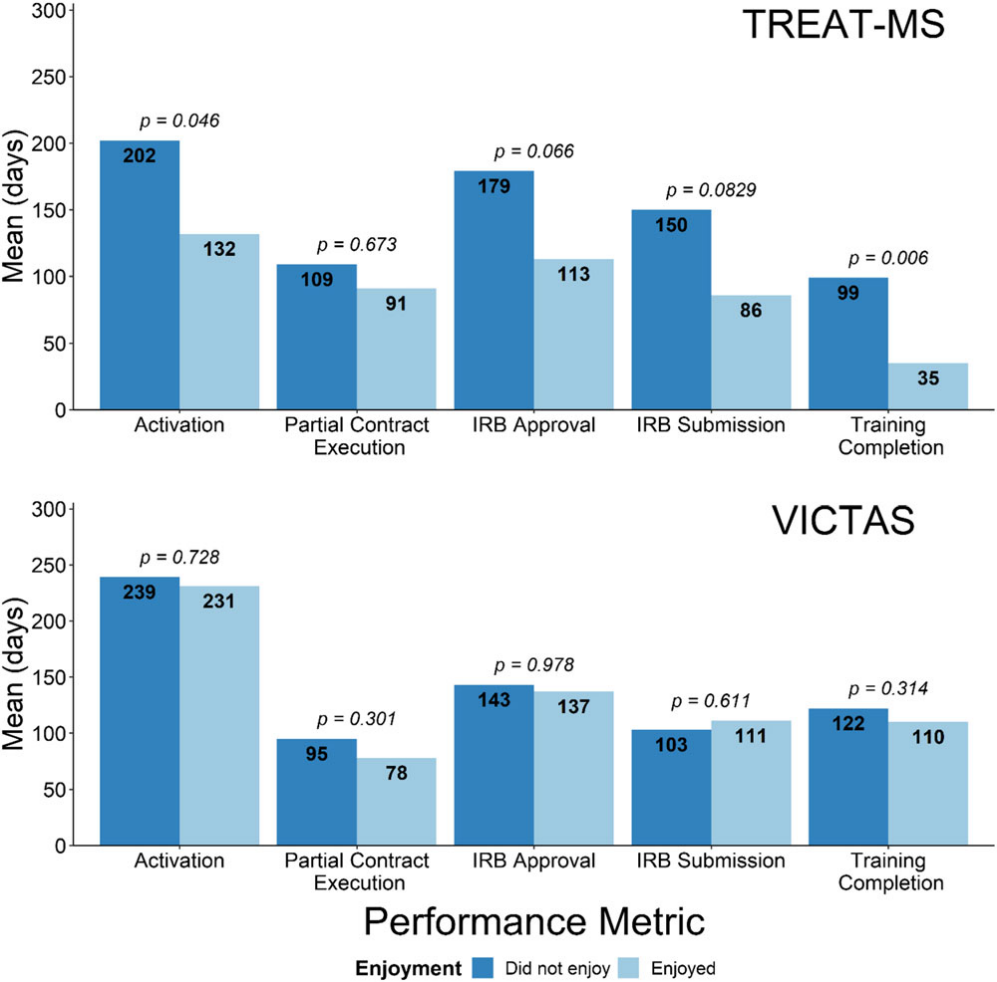
Mean differences in various start-up timeline metrics between those who enjoyed or did not enjoy the Mt. Everest game by study. In TREAT-MS, those who enjoyed playing the game performed better, on average, in all metrics; shorter time to activation and training completion were significant. Differences in mean completion times in VICTAS were small.

In the TREAT-MS trial, overall game enjoyment appeared to be an important moderator of performance across most gaming metrics analyzed (Figs. 3 and 4). In TREAT-MS, those who enjoyed playing the game, on average, had higher Mt. Everest scores (Fig. 3) and accomplished their start-up tasks more quickly (activation, partial contract execution, IRB submission, IRB approval, and training) (Fig. 4). The association between enjoyment with gaming and mean activation time and training completion time was statistically significant; the other metrics trended in the same direction and, importantly, provided raw mean differences that are very trial-relevant. For example, those who enjoyed the game completed contract execution 18 days faster, IRB submission 64 days faster, and IRB approval 66 days faster than those who did not enjoy the game. In the VICTAS trial, enjoyment had little effect on performance.

**Fig. 5.**
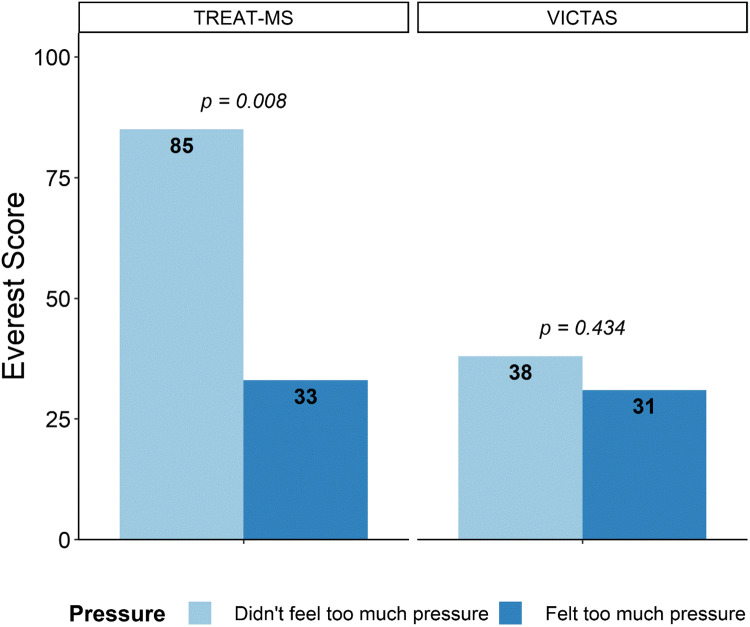
Mean differences in Mt. Everest Score between those who did or did not feel too much pressure playing the Mt. Everest game. Lack of feeling pressure was significantly associated with better Mt. Everest performance in TREAT-MS but not in VICTAS.

**Fig. 6.**
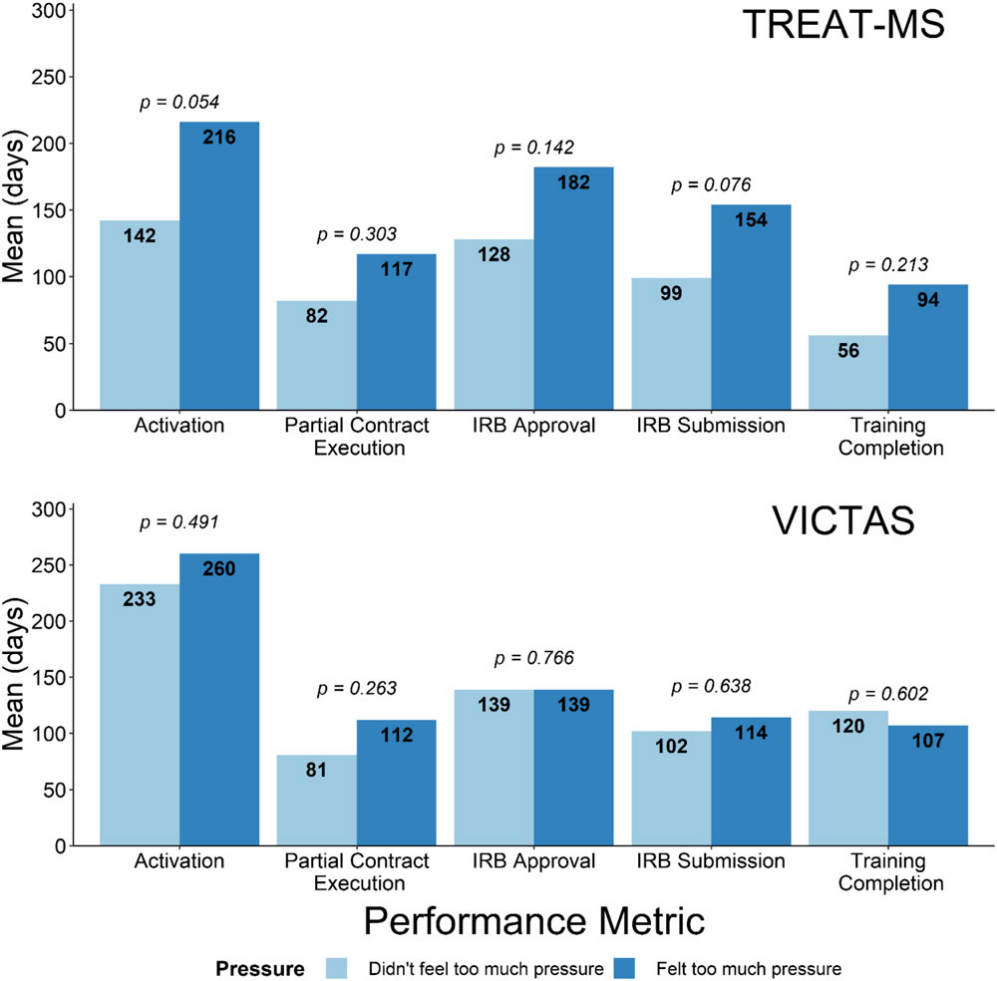
Mean differences in various start-up timeline metrics between those who did or did not feel too much pressure playing the Mt. Everest game by study. In TREAT-MS, those not feeling pressured performed better across all metrics; no result was significant. Differences in mean completion times in VICTAS were small.

Pressure also seemed to be an influential marker of performance in TREAT-MS but less so in VICTAS (Figs. 5 and 6). In TREAT-MS, those who felt the game did not apply too much pressure were much quicker to complete all their start-up objectives than those who felt too much pressure from the game (Fig. 6). However, this trend did not hold in the VICTAS trial. Mean Mt. Everest scores between pressured and not pressured were roughly similar, 38 and 31 respectively (see Fig. 5), and there was no difference in mean IRB approval time and training completion time between groups (see Fig. 6).

## Discussion

Games appear in many facets of our lives as a source of entertainment, relationship-building, and motivation. In today’s digital age, games can evolve and expand, bringing typical game elements and mechanics to ordinarily non-game contexts to motivate and engage people.

The Mt. Everest gamification experiences suggest that a game, based on the ordinary metrics of a trial start-up, can be well-designed to assign progress as standings to praise and encourage site teams and track well with actual performance. Across both trials, scores were found to be reflective of overall site performance in each of the metrics analyzed. This, in combination with improvements in cycle times for those who enjoyed the game, makes gamification a useful tool in future trials.

This is encouraging for trial sponsors and stakeholders, game developers, and those who are thinking about connecting clinical trial tasks to a points or scoreboard system. Continued development of games, using electronic trial management tools that provide clinical trial metrics and benchmark data, will support better management of clinical trials and provide sets of connections for gamification [7]. Within a trial, there are many possible elements that can be tracked and gamified without undue focus on the finish line. Game leaderboards can be incorporated into EDC systems with multiple metrics to recognize and applaud well-performing site teams across a variety of metrics rather than one metric, such as activation time or the number of enrollments. Depending on the complexity of the trial protocol and the capabilities of trial tools, such as the EDC, gaining points across many small achievements in trial cycles can reflect the overall performance in critical performance areas yet allow site teams to compete for the lead in one area or another.

Gamification provides the visual and communal opportunity to follow productivity; it allows a site team to track progress towards its own goals while comparing itself with other site teams and the overall goals of the trial. It also provides the opportunity to publicly praise performance and share desired behaviors with those lagging behind. If gamification can improve cycle times, it could stand to reason that using games would be most effective if coupled with ways to convert those who do not enjoy gamifying the workplace into those who do. Although these games are passive and participation is voluntary, if a game standing has a reverse effect on enjoyment and motivation, site teams could be offered opt-in or opt-out of leaderboard postings that release site standings to public view. This would naturally separate site teams into groups that want to view the game standings from those who do not, and it could allow exploration of the question: “Does willingness to play a game in a clinical trial affect performance in that trial?”

Gamification has been shown to be incredibly effective in the workplace [4,8]. When creating a game in a context where games are not usually played (such as among serious-minded research professionals), it may be crucial to design into game rewards activities that are meaningful to research productivity and trial progress – to make a difference in science as well as the game. The benefits of gamifying clinical trial activities – particularly when site teams feel disengaged and unmotivated – are that gamifying trial metrics and goals, in and of itself, encourages engagement. Additionally, focusing on game standings, if a competitive spirit is sparked, can be motivating and lead to an increase in quality and productivity and a sense of satisfaction with meeting the goals of a trial [4]. While turning tasks into games in these instances does not alleviate burdens, it turns burdens into opportunities for recognition through friendly competition.

## Limitations

Since this is the first study of the use of gamification for study start-up and execution, a larger number of trial experiences with prospectively gathered data will be needed to better define the benefits or unwanted effects of gamifying site activation. For future tests of games, it will be important to design measures that prospectively capture and correlate gaming in the clinical trial workplace with better satisfaction doing the work at hand, improved performance, and enjoyment of the game itself. For now, game utilization should be approached with equipoise.

The analysis regarding enjoyment was a retrospective inspection of limited available survey data. These surveys were not designed with the intent of being part of a quantitative analysis and were meant originally for internal quality improvement. Furthermore, the yes/no nature of the survey questions produced a less predictive analysis and future surveys will use a continuous scale from 0 to 10. From the limited nature of the surveys, it is unclear in which direction the causative arrow points: did enjoyment cause good performance or good performance cause enjoyment? In the former, it might suggest that players who enjoy competition might be more motivated to push various tasks to completion for the sake of the game in addition to, or even instead of, usual work-ethic motivations. In this case, future game designers should focus on making the experience of the game itself more enjoyable, improving engagement with the theme, and ensuring that rules are intuitive, challenging, and rewarding to the players.

It is unclear if site experience in executing trial activities plays a confounding role in overall game performance and/or enjoyment. The coordinating center built the Mt. Everest game within a protocolized start-up sequence that was standard across sites in the trial. The standardization limited the variability among site resources by assigning a site navigator to persistently engage and assist site teams with start-up tasks; yet, we recognize differences cannot be entirely eliminated. Skilled and established research teams with ample resources to devote to the trial may perform familiar tasks at a high level with or without a game (and vice versa for inexperienced sites). This relationship between site experience and level of further improvement after gamifying tasks is unknown and requires further exploration.

There has not been a cost analysis of transforming familiar trial metrics into scoring displays or creating graphics for online sharing in the form of a game. The Mt. Everest game was designed and launched by coordinating center staff intermediately knowledgeable in computer skills. An automated EDC makes the game management easier, but external or professional vendors are not required. Once a game theme and scoring system are developed for a first trial, it can be turnkey for subsequent use. No effort is required of the site teams, except to visit the display sites and attend regularly scheduled trainings and meetings. Site teams play the game simply by performing their trial responsibilities.

## Next Steps

This retrospective examination of available data from two gaming experiences may be first-of-kind use in clinical trials; no other publications on engaging trial personnel could be found. Its novel use has piqued the interest of a larger federation of coordinating centers within the Trial Innovation Network to explore if it really works and, if so, how to use gamification to improve trial performance. The Trial Innovation Network gamification working group mission is to *incorporate gaming innovations into clinical trial operations for networks to enhance site engagement, using competition as team building to improve task completion enjoyment and ultimately improve performance*.

In this very new endeavor, more data and surveys on clinical trial gamification will be needed. As one next step, a Gamification Toolkit on how to gamify clinical trial activities is posted to a Trial Innovation Network website (https://trialinnovationnetwork.org/) Toolbox Page to encourage more trialists to consider the use of games, thereby stimulating more data generation and published results. Isolating the effect of gamification on trial outcomes and improved satisfaction among site teams will depend on a larger sampling of prospective data using well-defined, hypothesis-driven statistical analysis plans.
